# Customized glenoid implants in reverse total shoulder arthroplasty: assessing revision rate and causes of failure in the Australian Joint Registry

**DOI:** 10.1016/j.jsea.2026.100098

**Published:** 2026-09-29

**Authors:** Megan C. Banks, Stefan Bauer, Peiyao Du, David RJ. Gill, William G. Blakeney

**Affiliations:** a Faculty of Medicine, Nursing, and Midwifery, and Health Sciences, University of Notre Dame Australia, Fremantle, WA, Australia; b Department of Orthopaedic Surgery, Royal Perth Hospital, Perth, WA, Australia; c School of Surgery, University of Western Australia, Perth, WA, Australia; d South Australian Health and Medical Research Institute (SAHMRI), Adelaide, SA, Australia; e Australian Orthopaedic Association National Joint Replacement Registry (AOANJRR), Adelaide, SA, Australia

**Keywords:** Primary shoulder arthroplasty, Reverse shoulder arthroplasty, Customized implant, 3D-printed, Revision rates, Revision shoulder arthroplasty

## Abstract

**Background:**

Severe bone loss presents as a challenge for surgeons during a reverse total shoulder arthroplasty (rTSA). Customized three-dimensional–printed glenoid implants have been developed to address complex deformities, but comparative data on their survivorship remain limited. This study examined demographics, revision rates and causes in patients undergoing primary or revision rTSA using custom three-dimensional–printed glenoid components compared to standard implants.

**Methods:**

This was a retrospective national registry study of patients undergoing primary and revision rTSA. Cohorts were defined as (1) primary rTSA and (2) first revision rTSA, each stratified by use of either custom glenoid implants (primarily Zimmer-Biomet and Lima/Enovis) or standard prostheses. Revision was analyzed using Kaplan–Meier estimates of survivorship and hazard ratios (HRs) from Cox proportional hazard models, adjusting for age and gender. The reasons for revision were compared as a secondary outcome measure. Patient-reported outcome measures were not analyzed.

**Results:**

Of 49,230 primary rTSA and 1,597 first revision rTSA, there were 157 primary and 31 first revision custom rTSA procedures. The cumulative percent revision for primary custom rTSA was 7.1% (95% confidence interval 3.8–13.2) at 4 years. Following first revision rTSA using a custom glenoid, the cumulative percent revision for second revision was 21.0% (95% confidence interval 9.0–46.0) at 5 years. The custom rTSA group had a higher rate of revision compared to standard implants (HR = 1.84 [1.02, 3.33], *P* = .043), whereas no statistically significant difference was observed in the revision setting (HR = 1.15 [0.48, 2.80], *P* = .750) or when restricted to patients with more severe deformity (Walch morphology type C) (HR = 1.92 [0.68, 5.42], *P* = .219). The causes of revision tended to be consistent between groups, being instability/dislocation (n = 4 [36.4%] vs. n = 600 [32.6%]), infection, and loosening (n = 3 [27.3%] vs. n = 300 [16.3%]), noting that statistical comparison was limited by small numbers in the custom cohort.

**Conclusion:**

In this national registry study, customized glenoid implants in primary rTSA showed a higher overall revision rate than standard implants, but this appears driven by case complexity: customized implants were predominantly used for severe deformity (notably Walch C). When analysis was limited to Walch C glenoids, revision risk was not significantly different between customized and standard implants. Failure modes were similar across groups—instability/dislocation, loosening, and infection were the leading causes—and second revision rates after revision rTSA did not differ, acknowledging small numbers. Further case-matched studies with better deformity quantification and patient-reported outcome measures are needed to define optimal indications and long-term value of customized implants.

The reverse total shoulder arthroplasty (rTSA) was developed in the late 1980s,^11^, ^8^ initially for degenerative arthropathy and rotator cuff tear, though its indications have expanded vastly.^12^ As of 2024, the rTSA accounts for 87.4% of primary shoulder replacements in Australia, and is reported as having excellent clinical outcomes, patient-reported outcome measures (PROMs), and lower revision rates compared to traditional, anatomic total shoulder arthroplasty.^21^ According to the Australian Orthopaedic Association National Joint Replacement Registry (AOANJRR), there is a 5.1%-6.5% 10-year cumulative revision rate of primary rTSA.^21^ However, there are instances whereby standard rTSA reaches its limitations, namely, in cases whereby patients have inadequate bone stock to allow for grafting, as is sometimes the case in advanced degenerative disease or revision surgery.^16^, ^4^

Personalized medicine has grown immensely in orthopedics, exemplified by imaging-based navigation systems to optimize alignment, and patient-specific instruments.^7^ More recently, patient-specific customized implants have been employed to match deformity, bone loss, and defects, as well as to improve the accuracy of implantation and outcomes in arthroplasty.^2^ Such implants, and the associated revision rates and outcomes, have been described most often in knee and hip arthroplasty, with mixed results.^1^, ^18^, ^19^ However, this technology is more recently being utilized in shoulder arthroplasty. Such custom components can be manufactured to match the deformed or deficient glenoid, and they often incorporate microstructural porosity engineering to facilitate osseointegration, with the goal of accurately reconstructing premorbid anatomy and improving stability.^16^, ^4^

There are limited number of studies about customized shoulder arthroplasty evaluating revision rates and clinical outcomes.^16^, ^4^, ^2^, ^17^, ^10^ These studies have reported significant post-operative improvements in PROMs and functional scores.^5^, ^13^, ^6^, ^15^, ^3^, ^14^ However, to date, there is limited reporting on complication and revision rates with extended follow-up. One systematic review included 4 studies and reported a complication and revision rate over 2 years as 21% and 10.5%, respectively (*n* = 46).^9^ Though another multi-surgeon study reported a revision rate of 6.9% over a 2-year follow-up period (*n* = 42).^16^ Moreover, in a 5-year follow-up study, the revision rate was as high as 16% (*n* = 37).^5^ While these studies have shed some light on the safety and efficacy of customized implants in rTSA, they have been largely inconsistent, relatively small, and often single surgeon studies. Most notably, there have been neither studies to have considered a comparison to a control group nor any that have considered the degree of glenoid deformity in statistical analyses.

As such, the purpose and primary aim of this study was to examine revision rates, or survivorship, of customized implants in a large group of patients who underwent rTSA, compared to those with standard implants. A secondary aim was to assess the leading causes of revision in custom implants. Moreover, the study aimed to contribute the first Australian perspective on the revision rate and leading causes of revision surgery following customized implant rTSA.

## Materials and methods

### Study design and data collection

This was a retrospective national registry study that utilized quantitative analysis to consider patients undergoing rTSA. Data were obtained from the AOANJRR national audit. The specific data collection and recruitment strategies are outlined below.

## Australian Orthopaedic Association National Joint Replacement Registry

### Registry background

The AOANJRR began collecting data on shoulder arthroplasty procedures since April 2004, and the registry has documented 97.1% of these procedures Australia-wide since November 2007. These data are externally validated against patient-level data provided by all Australian state and territory health departments. A sequential, multi-level matching process is used to identify any missing data, which are subsequently obtained by follow-up with the relevant hospital. Each month, in addition to internal validation and data quality checks, all primary procedures are linked to any subsequent revision involving the same patient, joint, and side. Data are also matched biannually to the Australian National Death Index data to identify patients who have died. The AOANJRR began collecting the American Society of Anesthesiologists score in 2012 and body mass index in 2015, and glenoid morphology, as classified by Walch,^22^ in 2017.

### Study population

The study population included all primary and revision, stemmed rTSAs performed for all diagnoses between April 16, 2004, and December 31, 2022.

Two analytically distinct cohorts were defined as follows:

1. Primary rTSA cohort:

All primary rTSA procedures, stratified by use of either a customized glenoid implant or a standard glenoid implant.

2. Revision rTSA cohort:

All first revision rTSA procedures with a known primary procedure, stratified by use of either a customized glenoid implant or a standard glenoid implant at the time of revision.

Custom glenoid implants included all patient-specific designs recorded in the AOANJRR, predominantly iterations of 2 implants: the Custom Comprehensive Reverse Glenoid Baseplate (Zimmer-Biomet) and Custom Made Glenoid Ti6Al4V (Lima/Enovis) (Supplementary Tables A1 and A2). All other glenoid components were classified as standard implants. No implant models were excluded.

### Study outcomes

The primary outcome differed by cohort as follows:

• In the primary rTSA cohort, the primary end point was time to first revision for any reason.
• In the revision rTSA cohort, the primary end point was time to second revision (ie, re-revision) for any reason.

Revision was defined according to AOANJRR criteria as any subsequent procedure involving removal, replacement, or addition of 1 or more prosthetic components.

Secondary outcomes included descriptive analysis of the recorded causes of revision, including instability/dislocation, infection, and loosening.

PROMs were not analyzed due to insufficient completion rates in the custom glenoid cohorts.

### Ethics approval

The AOANJRR was approved by the Commonwealth of Australia as a Federal Quality Assurance Activity (F2022L00986) Part VC, under section 124X of the Health Insurance Act 1973 and Part 10 of the Health Insurance Regulations 2018. All AOANJRR studies are conducted in accordance with ethical principles of research (the Helsinki Declaration II).

### Statistical analysis

Kaplan–Meier estimates of survivorship were used to report the time to second revision, with censoring at the time of death and closure of the dataset at the end of December 2022. The unadjusted cumulative percent revision, with 95% confidence intervals (CIs), was calculated using unadjusted point wise Greenwood estimates. Age and gender adjusted hazard ratios (HRs) were calculated from Cox proportional hazard models to compare the rate of second revision between groups. The assumption of proportional hazards was checked analytically for each model. If the interaction between the predictor and the log of time was statistically significant in the standard Cox model, then a time varying model was estimated. Time points were selected based on the greatest change in hazard, weighted by a function of events. Time points were iteratively chosen until the assumption of proportionality was met, and HRs were calculated for each selected time-period. For the current study, the HR was calculated over the entire follow-up period.

## Results

### Part 1 – characterizing the dataset

#### Number of individuals with custom implants in the Australian Orthopaedic Association National Joint Replacement Registry cohorts

There were 157 patients who underwent a primary rTSA with a custom implant and 31 individuals who underwent a revision rTSA with a custom implant in the AOANJRR cohort (Fig. 1).

**Figure 1:**
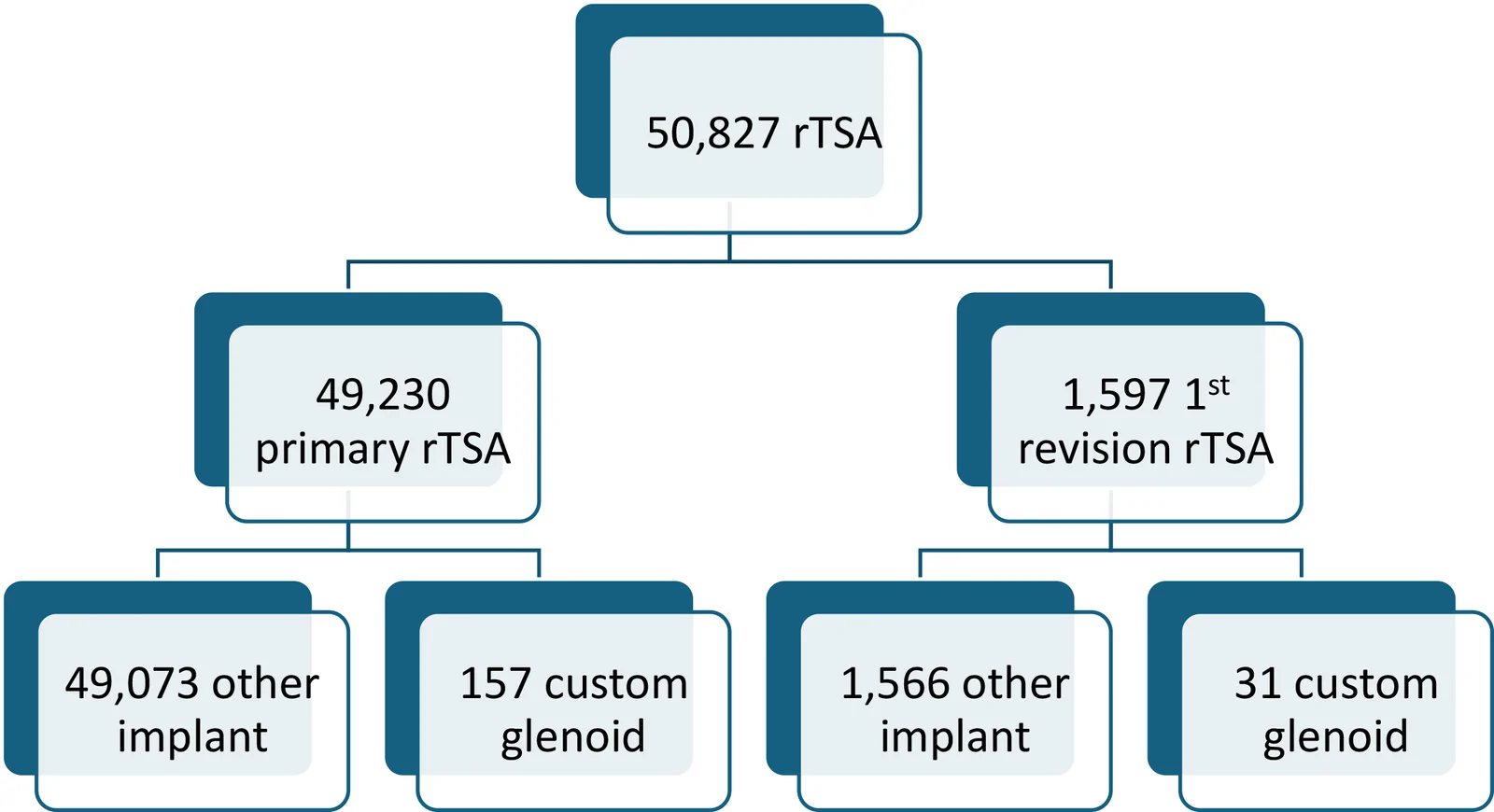
Number of participants in each group within the AOANJRR considered in this study. *AOANJRR*, Australian Orthopaedic Association National Joint Replacement Registry.

#### Demographic differences between custom and other implant groups in the primary reverse total shoulder arthroplasty Australian Orthopaedic Association National Joint Replacement Registry cohort

In individuals undergoing their primary procedure, custom glenoid participants were followed up for 3.1 years ( ± 2.2 years) on average, shorter than those with other standard implants (Table I).

**Table I:** Summary of primary total stemmed reverse shoulder replacement by glenoid class (all diagnoses)

Variable	Custom glenoid	Other implants	Total
Follow-up years			
Mean ± SD	3.1 ± 2.2	4.1 ± 3.2	4.1 ± 3.2
Median (IQR)	3 (1.4, 4.4)	3.4 (1.6, 6)	3.4 (1.6, 5.9)
Minimum	0	0	0
Maximum	12.7	16.9	16.9
Age			
Mean ± SD	71.4 ± 10.7	73.7 ± 8.2	73.7 ± 8.2
Median (IQR)	73 (67, 78)	74 (69, 79)	74 (69, 79)
Age group			
<55	8 (5.1%)	803 (1.6%)	811 (1.6%)
55-64	23 (14.6%)	5,344 (10.9%)	5,367 (10.9%)
65-74	62 (39.5%)	19,232 (39.2%)	19,294 (39.2%)
≥75	64 (40.8%)	23,694 (48.3%)	23,758 (48.3%)
Gender			
Male	64 (40.8%)	18,525 (37.7%)	18,589 (37.8%)
Female	93 (59.2%)	30,548 (62.3%)	30,641 (62.2%)
ASA score∗			
1	5 (3.3%)	1,218 (2.9%)	1,223 (2.9%)
2	47 (31.1%)	17,061 (40.6%)	17,108 (40.5%)
3	93 (61.6%)	22,406 (53.3%)	22,499 (53.3%)
4	6 (4%)	1,377 (3.3%)	1,383 (3.3%)
5		5 (0%)	5 (0%)
BMI†			
Underweight (<18.50)	1 (0.7%)	294 (0.8%)	295 (0.8%)
Normal (18.50-24.99)	33 (22.6%)	6,173 (17.2%)	6,206 (17.2%)
Pre-obese (25.00-29.99)	54 (37%)	12,372 (34.4%)	12,426 (34.4%)
Obese class 1 (30.00-34.99)	27 (18.5%)	9,668 (26.9%)	9,695 (26.8%)
Obese class 2 (35.00-39.99)	20 (13.7%)	4,755 (13.2%)	4,775 (13.2%)
Obese class 3 (≥40.00)	11 (7.5%)	2,722 (7.6%)	2,733 (7.6%)
Primary diagnosis			
Fracture	2 (1.3%)	7,507 (15.3%)	7,509 (15.3%)
Instability	3 (1.9%)	494 (1%)	497 (1%)
Osteoarthritis	93 (59.2%)	21,246 (43.3%)	21,339 (43.3%)
Osteonecrosis	2 (1.3%)	544 (1.1%)	546 (1.1%)
Other	1 (0.6%)	11 (0%)	12 (0%)
Other inflammatory arthritis	5 (3.2%)	234 (0.5%)	239 (0.5%)
Rheumatoid arthritis	16 (10.2%)	829 (1.7%)	845 (1.7%)
Rotator cuff arthropathy	34 (21.7%)	17,962 (36.6%)	17,996 (36.6%)
Tumor	1 (0.6%)	246 (0.5%)	247 (0.5%)
Morphology‡			
A	18 (15.8%)	18,144 (68.6%)	18,162 (68.3%)
B	27 (23.7%)	7,060 (26.7%)	7,087 (26.7%)
C	69 (60.5%)	1,262 (4.8%)	1,331 (5%)
Total	157	49,073	49,230

*SD*, standard deviation; *IQR*, interquartile range; *ASA*, American Society of Anesthesiologists; *BMI*, body mass index (kg/m^2^).

∗ Excludes 7,012 procedures with unknown ASA score.

† Excludes 13,100 procedures with unknown BMI.

‡ Excludes 22,650 procedures with unknown morphology.

Individuals with primary procedure custom implants were younger, on average (71.4 years), compared with those with standard implants (Table I). While the primary custom glenoid groups were primarily made up of individuals aged 65 to 74 years (39.5%), they had a higher proportion of individuals who were less than 64 years when compared to the other implant group (19.7% vs. 12.5%).

When comparing the cohort with custom implants to all others, there were vast similarities in their groupings of gender, American Society of Anesthesiologists level, and body mass index (Table I).

Of note, there were differences in the primary diagnosis that caused the requirement of rTSA (Table I). The leading primary diagnosis was osteoarthritis in both instances (59.2% and 43.3%, respectively). However, in the custom group, rheumatoid arthropathies and other inflammatory arthropathies were over-represented (13.4%), and fracture and instability were under-represented.

Finally, there were notable differences in the glenoid morphology seen in the custom group when compared to all other implants. Classified by Walch, the glenoid is categorized into neutral with “symmetric erosion…explained by the absence of subluxation” (A), posterior subluxation causing “asymmetric load against the glenoid “and is “implicated in the development of primary glenohumeral arthritis” (B), and >25° retroversion characteristically being “primarily of dysplastic origin and explained the early event of osteoarthritis” (C).^22^ The majority of custom glenoid group, with known morphology, was made up of individuals with a morphology C (60.5%), a stark difference to the other implant group, where only 4.8% of participants had morphology C. The majority of glenoid morphologies seen in the non-custom glenoid group were type A (68.6%) (Table I).

#### Demographic differences between custom and other implant groups in the revision reverse total shoulder arthroplasty Australian Orthopaedic Association National Joint Replacement Registry cohort

In individuals undergoing their revision procedure, custom glenoid participants were followed up for 3.6 years ( ± 2.4 years) on average, shorter than those with other standard implants (Table II).

**Table II:** Summary of first glenoid revision shoulder replacement by glenoid class (all diagnoses)

Variable	Custom glenoid	Other implants	Total
Follow-up years			
Mean ± SD	3.6 ± 2.4	4.7 ± 3.7	4.6 ± 3.7
Median (IQR)	3.6 (1.6, 4.8)	4.1 (1.3, 7.3)	4.1 (1.3, 7.2)
Minimum	0	0	0
Maximum	9.5	15.9	15.9
Age			
Mean ± SD	69.7 ± 10	68.8 ± 9.8	68.8 ± 9.8
Median (IQR)	71 (62, 77)	70 (63, 76)	70 (63, 76)
Age group			
<55	3 (9.7%)	118 (7.5%)	121 (7.6%)
55-64	6 (19.4%)	361 (23.1%)	367 (23%)
65-74	10 (32.3%)	618 (39.5%)	628 (39.3%)
≥75	12 (38.7%)	469 (29.9%)	481 (30.1%)
Gender			
Male	16 (51.6%)	643 (41.1%)	659 (41.3%)
Female	15 (48.4%)	923 (58.9%)	938 (58.7%)
ASA score∗			
1	1 (3.3%)	43 (3.6%)	44 (3.6%)
2	8 (26.7%)	500 (41.4%)	508 (41%)
3	18 (60%)	629 (52%)	647 (52.2%)
4	3 (10%)	37 (3.1%)	40 (3.2%)
BMI†			
Underweight (<18.50)		6 (0.6%)	6 (0.6%)
Normal (18.50-24.99)	8 (30.8%)	153 (15.5%)	161 (15.9%)
Preobese (25.00-29.99)	10 (38.5%)	325 (33%)	335 (33.2%)
Obese class 1 (30.00-34.99)	5 (19.2%)	294 (29.9%)	299 (29.6%)
Obese class 2 (35.00-39.99)	2 (7.7%)	134 (13.6%)	136 (13.5%)
Obese class 3 (≥40.00)	1 (3.8%)	72 (7.3%)	73 (7.2%)
Revision diagnosis			
Arthrofibrosis		15 (1%)	15 (0.9%)
Dissociation		28 (1.8%)	28 (1.8%)
Fracture	1 (3.2%)	88 (5.6%)	89 (5.6%)
Glenoid erosion	5 (16.1%)	154 (9.8%)	159 (10%)
Heterotopic bone		1 (0.1%)	1 (0.1%)
Implant breakage glenoid		16 (1%)	16 (1%)
Implant breakage glenoid insert		7 (0.4%)	7 (0.4%)
Implant breakage head		5 (0.3%)	5 (0.3%)
Incorrect sizing		6 (0.4%)	6 (0.4%)
Infection	3 (9.7%)	100 (6.4%)	103 (6.4%)
Instability/dislocation	5 (16.1%)	274 (17.5%)	279 (17.5%)
Loosening	12 (38.7%)	373 (23.8%)	385 (24.1%)
Lysis	1 (3.2%)	25 (1.6%)	26 (1.6%)
Malposition		17 (1.1%)	17 (1.1%)
Metal related pathology		7 (0.4%)	7 (0.4%)
Osteonecrosis		1 (0.1%)	1 (0.1%)
Other		15 (1%)	15 (0.9%)
Pain	1 (3.2%)	122 (7.8%)	123 (7.7%)
Rotator cuff insufficiency	3 (9.7%)	304 (19.4%)	307 (19.2%)
tumor		3 (0.2%)	3 (0.2%)
Wear glenoid insert		5 (0.3%)	5 (0.3%)
Morphology‡			
A	2 (15.4%)	298 (70.4%)	300 (68.8%)
B	1 (7.7%)	97 (22.9%)	98 (22.5%)
C	10 (76.9%)	28 (6.6%)	38 (8.7%)
Total	31	1,566	1,597

*SD*, standard deviation; *IQR*, interquartile range; *ASA*, American Society of Anesthesiologists; *BMI*, body mass index (kg/m^2^).

∗ Excludes 358 procedures with unknown ASA score.

† Excludes 587 procedures with unknown BMI.

‡ Excludes 1,161 procedures with unknown Morphology.

Individuals with revision procedure custom implants were 1 year older, on average (69.7 years), compared with those with standard implants (Table II). The revision custom glenoid group were primarily made up of individuals aged 75 and older (38.7%), whereas the other implant group were primarily made up of individuals aged between 65-74 years (39.5%).

Of note, there were differences in the diagnosis that caused the revision rTSA (Table II). The leading revision diagnosis was loosening in both custom and standard implants (38.7% and 23.8%, respectively). In the custom group, the second most common revision cause was glenoid erosion and instability/dislocation, equally (16.1%, Table II). Whereas, in the other implant group, the second most common revision cause was rotator cuff insufficiency (Table II).

Finally, there were vast differences again seen in glenoid morphology of the custom group when compared to all other implants in revision rTSA. The majority of custom glenoid group was made up of individuals with a morphology C (76.9%), a large difference to the other implant group, where only 6.6% of participants had morphology C. The majority of glenoid morphologies seen in the non-custom glenoid group were type A (70.4%) (Table II).

### Part 2 – revision in reverse total shoulder arthroplasties in the Australian Orthopaedic Association National Joint Replacement Registry cohort

#### Higher rate of revisions in custom glenoid group in primary procedures, but not in revision

Within primary rTSA procedures, a higher rate of revision was observed in the custom glenoid group, which was significant in multi-variate analyses controlling for age and gender (Fig. 2, HR 1.84 [95% CI 1.02-3.33]; *P* = .043).

**Figure 2:**
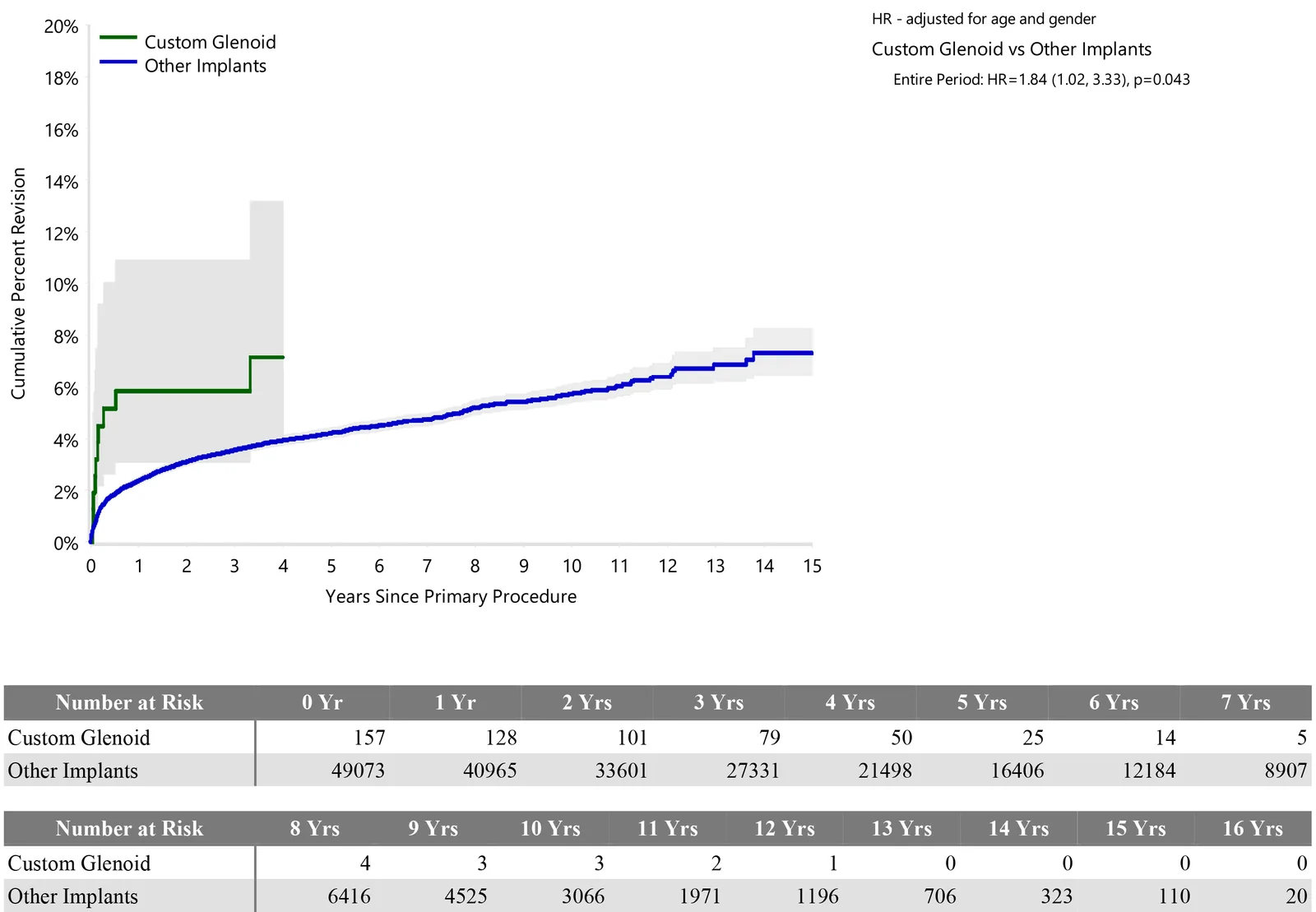
Cumulative percent revision of primary total stemmed reverse shoulder replacement by glenoid class.

However, within revision rTSA procedures, there was no significant difference between the rate of second revision in the custom and standard prosthesis groups, when controlling for age and gender (Fig. 3, HR 1.15 [95% CI 0.48-2.80]; *P* = .750).

**Figure 3:**
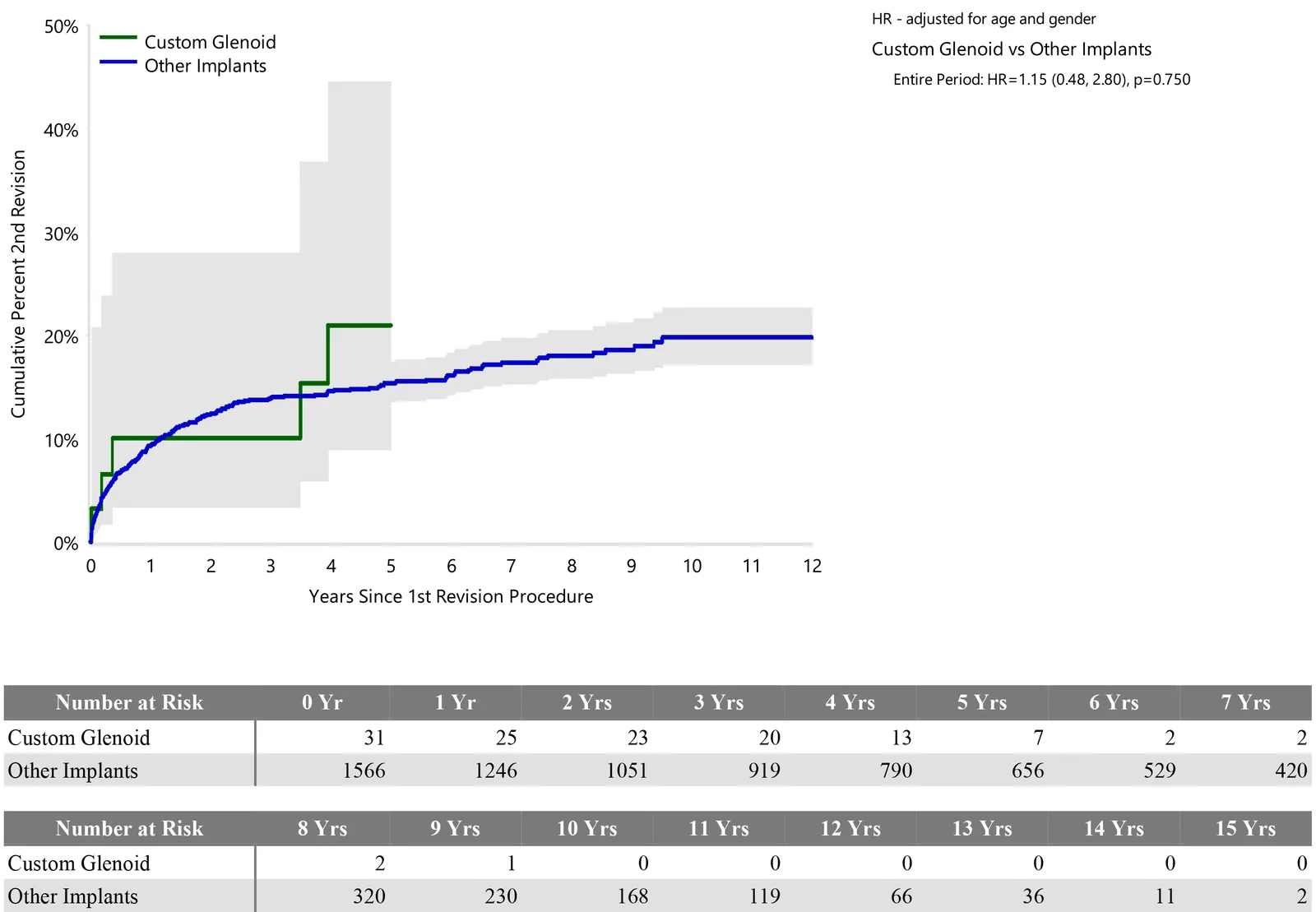
Cumulative percent second revision of first glenoid revision shoulder replacement by glenoid class.

#### In individuals with Walch morphology C, no significant difference seen in rate of revisions between custom and other implant groups in primary rTSA procedure

Due to the significant difference in proportion of morphology between custom glenoid groups and other implant groups within the primary rTSA procedures of the AOANJRR cohort, sub-analysis was restricted to primary procedures in individuals with an original glenoid morphology of Walch morphology C.

In these individuals with matched morphology type, there were no significant difference in rate of revision between custom glenoid or other implant groups (HR 1.92 [95% CI 0.68-5.42]; *P* = .219) after adjusting for age and gender (Fig. 4).

**Figure 4:**
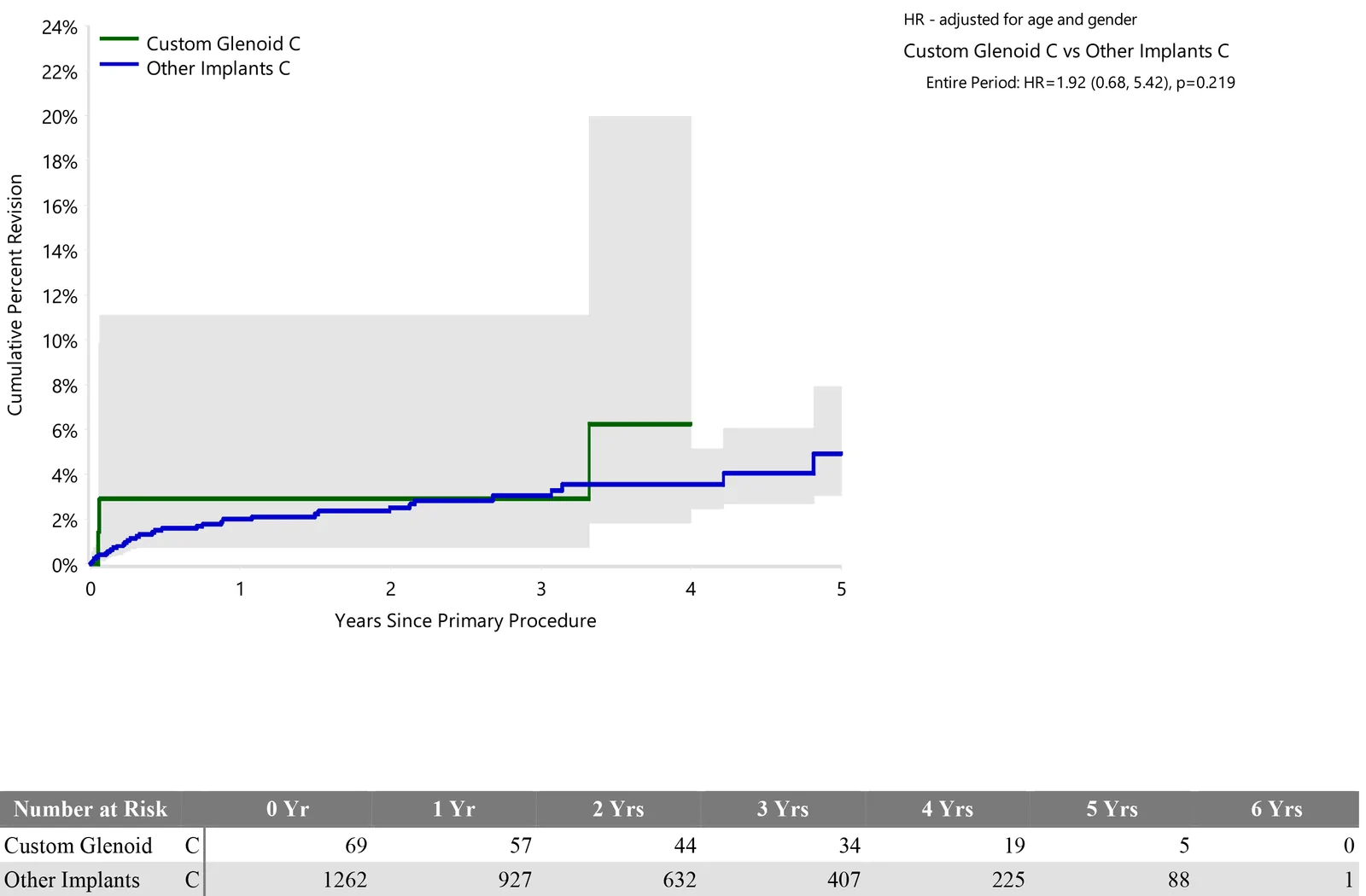
Cumulative percent revision of primary total stemmed reverse shoulder replacement by glenoid class and morphology.

### Part 3 - cause of revision in reverse total shoulder arthroplasty groups within the Australian Orthopaedic Association National Joint Replacement Registry cohort

#### Cause of revisions appears consistent between implant groups, in primary and revision procedures

The majority of primary revisions were due to instability and dislocation in both the custom glenoid group and the other implant group (Table III, n = 4 [36.4%] and n = 600 [32.6%] of revisions, respectively). Proportions suggest that the top 3 causes of revision may be consistent regardless of type of implant used; however, due to the small sample size, statistical analyses between groups were not possible.

**Table III:** Revision diagnosis of primary total stemmed reverse shoulder replacement by glenoid class (all diagnoses) (follow-up limited to 12.7 y)

Revision diagnosis	Custom glenoid			Other implants		
	Number	% Primaries revised	% revisions	Number	% Primaries revised	% revisions
Instability/dislocation	4	2.5	36.4	600	1.2	32.6
Infection	2	1.3	18.2	454	0.9	24.7
Loosening	3	1.9	27.3	300	0.6	16.3
Fracture				208	0.4	11.3
Dissociation	2	1.3	18.2	64	0.1	3.5
Pain				37	0.1	2.0
Lysis				24	0.0	1.3
Arthrofibrosis				21	0.0	1.1
Malposition				21	0.0	1.1
Implant breakage glenoid				14	0.0	0.8
Incorrect sizing				12	0.0	0.7
Metal related pathology				12	0.0	0.7
Heterotopic bone				6	0.0	0.3
Rotator cuff insufficiency				6	0.0	0.3
Wear humeral cup				6	0.0	0.3
tumor				4	0.0	0.2
Implant breakage glenoid insert				3	0.0	0.2
Implant breakage humeral				3	0.0	0.2
Wear glenoid insert				2	0.0	0.1
Glenoid erosion				1	0.0	0.1
Synovitis				1	0.0	0.1
Other				42	0.1	2.3
N revision	**11**	**7.0**	**100.0**	**1841**	**3.8**	**100.0**
N primary	**157**			**49,073**		

This table is restricted to revisions within 12.7 y for all groups to allow a time-matched comparison of revisions.

Moreover, the majority of second revisions were also due to instability and dislocation in both the custom glenoid group and other implant group (Table IV, n = 3 [60.0%] and n = 77 [33.5%] of revisions, respectively).

**Table IV:** Revision diagnosis of first glenoid revision shoulder replacement by glenoid class (all diagnoses) (follow-up limited to 9.5 y)

Revision diagnosis	Custom glenoid			Other implants		
	Number	% primaries revised	% revisions	Number	% primaries revised	% revisions
Instability/dislocation	3	9.7	60.0	77	4.9	33.5
Loosening	1	3.2	20.0	66	4.2	28.7
Infection				38	2.4	16.5
Fracture	1	3.2	20.0	12	0.8	5.2
Dissociation				9	0.6	3.9
Rotator cuff insufficiency				6	0.4	2.6
Pain				4	0.3	1.7
Arthrofibrosis				3	0.2	1.3
Implant breakage glenoid insert				2	0.1	0.9
Wear humeral cup				2	0.1	0.9
Glenoid erosion				1	0.1	0.4
Heterotopic bone				1	0.1	0.4
Implant breakage glenoid				1	0.1	0.4
Implant breakage head				1	0.1	0.4
Implant breakage humeral				1	0.1	0.4
Malposition				1	0.1	0.4
Osteonecrosis				1	0.1	0.4
Wear glenoid				1	0.1	0.4
Wear glenoid insert				1	0.1	0.4
Other				2	0.1	0.9
N revision	**5**	**16.1**	**100.0**	**230**	**14.7**	**100.0**
N primary	**31**			**1,566**		

This table is restricted to revisions within 9.5 y for all groups to allow a time-matched comparison of revisions.

## Discussion

Customized, three-dimensional–printed glenoid implants are increasingly utilized in rTSA for managing severe glenoid bone loss, revision cases, and anatomical deformities not addressable with standard implants.^16^, ^4^, ^2^, ^17^, ^10^ These implants offer a patient-specific solution, often designed from computed tomography imaging, with screw trajectories, back-side conformations, and porous surfaces tailored to optimize fixation and promote osseointegration.^16^, ^4^ However, until recently, most evidence supporting their use has been limited to small case series and single-surgeon experiences. Although these studies have evaluated the results of customized rTSA, none had made statistical comparison to a control group, nor had any considered the degree of glenoid deformity in statistical analyses.

In the present study—the largest to date using national registry data—we observed a significantly higher overall revision rate following primary rTSA with custom implants compared to standard components. This finding aligns with several previous investigations that noted variable but often elevated early revision rates.^16^, ^5^, ^9^ Such a result was expected, as these custom rTSAs are typically reserved for those individuals who have the most difficult pathology to reconstruct, such as a severe deformity or low bone stock. Our data support this interpretation. Among patients receiving customized implants, 60.5% had Walch type C glenoid morphology, which is classified as >25° retroversion and often caused by profound posterior erosion and retroversion. In contrast, only 4.8% of those with standard implants had this morphology. When the analysis was limited to Walch type C cases, the revision risk between custom and standard implant groups was no longer statistically different. It is notable that a higher revision rate was not seen in this sub-group analysis. Underlying case difficulty, bone stock, and glenoid morphology often vastly differ in cases where custom prostheses are utilized when compared to standard prosthesis group, which may contribute revision rates. However, this study was limited to utilizing Walch classification system and low numbers in this sub-group analysis limits the ability to reliably draw conclusions. As such, we suggest future studies that have higher numbers and that closely characterize the contribution that case complexity makes to revision rate, when compared to prostheses type, as further studies that specifically case-match may reveal more reliable data on survival rate in these custom cases.

While prior studies have reported favorable short-term clinical outcomes with custom implants, including improvements in range of motion, pain, and patient-reported outcomes, long-term durability remains a concern.^16^, ^5^, ^9^ Rashid et al^16^ reported on 41 patients who underwent rTSA with a single custom implant platform (Lima) to manage severe glenoid bone loss, noting significant clinical improvement at a minimum of 2-year follow-up, but with a 10% failure rate, with humeral loosening being the most common issue. Chammaa et al^5^ also demonstrated significant functional improvement at a mean follow-up of 5 years in their study of 37 patients looking at another custom implant platform (Stanmore Implants Worldwide, Elstree, UK), with a revision rate of 16% due to either loosening, fracture, or dislocation. Our registry findings are consistent with these reports: instability/dislocation, infection, and loosening were the most frequent causes of revision for both custom and standard implants. This suggests that the mode of failure does not differ substantially between implant types, despite differences in the patient populations.

Interestingly, there was no significant difference in second revision rates between custom and standard implant groups in revision rTSA. This may be due to the fact that revision cases are more commonly difficult, potentially reducing the confounder of case difficulty. However, this sub-group analysis was limited by small sample size and should be interpreted cautiously. Ongoing data accrual will be essential to assess longer-term outcomes in this cohort.

Our findings underscore the need for a nuanced evaluation of custom glenoid technology. As three-dimensional printing becomes more cost-effective and widely available, the potential role of customized implants may expand beyond salvage cases to more routine arthroplasty. However, prospective studies are required to better understand their value in terms of cost-effectiveness, functional outcomes, and radiographic integration compared to augmented or bone-grafted standard implants.

### Limitations

Our study has several limitations. Firstly, the decision to revise surgery is complex and involves several patient-surgeon factors, and this cannot be accounted for in analyses. Secondly, given the advanced age of the cohort and the age imbalance between groups, death may represent an important competing risk for second revision. As death was treated as a censoring event in the Kaplan–Meier analysis, the estimated cumulative probability of revision may be overestimated. Thirdly, not all morphologies, primary diagnoses or causes of revision could be considered due to the sample size, which limited the ability to draw conclusions about the role of disease severity in revision rate and to understand why custom rTSAs were revised at a higher rate. As is the nature of retrospective registry data, quantitative assessment of pre-operative bone volume and angle of deformity was not collected, and thus, we were unable to control for such factors in statistical analysis. Furthermore, the AOA joint registry only classifies glenoids by the original Walch classification (which has since been modified), and does not include the Sirveaux and Favard classification favored for cuff arthropathy.^20^ Without such pre-surgical severity information, comparison of control and custom group is imperfect and does not represent a case-matched cohort. As such, the registry bias and selection bias, particularly in the custom glenoid group, limits the reliability of the statistical comparisons made. Moreover, the analysis based on Walch classification is limited by time, as glenoid morphology was collected from 2017 onwards. Another limitation is that PROMs could not be analyzed in the registry group due to the small number of completed PROMs in the custom glenoid groups. This is significant as we cannot reliably quantify the number of poorly performing, painful implants. Finally, while the Registry cohort had a significant number of participants, sub-group analysis often had small numbers, limiting some comparisons to having no statistical analyses. Thus, a larger study assessing PROMs (as this study did not), as well as carefully characterizing pre-operative bone stock and deformity, is required to add to current understandings and to better case-match for reliable statistical comparison with limited selection and registry bias.

## Conclusion

In this national registry study, customized glenoid implants in primary rTSA showed a higher overall revision rate than standard implants. However, customized implants were predominantly used for severe deformity (notably Walch C). When analysis was limited to Walch C glenoids, revision risk was not significantly different between customized and standard implants. Notably, these groups are not case-matched; and we expect that those treated with custom glenoids are likely to have more severe bone loss and deformity. Case complexity may meaningfully contribute to revision rates, though the present study was unable to examine the difference that case complexity vs. prosthesis type contributes to revision rates.

Failure modes were similar across groups—instability/dislocation, loosening and infection were the leading causes—and second revision rates after revision rTSA did not differ, acknowledging small numbers. Further case-matched studies with better deformity quantification and PROMs are needed to define optimal indications and long-term value of customized implants, thought the current study has not ruled out the role of custom glenoids in managing complex glenoid deformities.

## Disclaimers:

Funding: No funding was disclosed by the authors.

Conflicts of interest: Dr Bauer is a paid consultant for Enovis and Stryker. Dr Blakeney is a paid consultant for Enovis and reports consultancy work/fees with Lima and Stryker. Any additional authors, their immediate family, and any research foundation with which they are affiliated have not received any financial payments or other benefits from any commercial entity related to the subject of this article.

## Ethics declaration

Written informed consent to take part in the study and to publish the article has been obtained from all participants or their legal representatives. The privacy rights of participants have been observed.

## Supplementary Data

Supplementary Appendices A1 and A2

## References

[1] Beit Ner E., Dosani S., Biant L.C., Tawy G.F. (2021). Custom implants in TKA provide no substantial benefit in terms of outcome scores, reoperation risk, or mean alignment: a systematic review. Clin Orthop Relat Res.

[2] Benignus C., Buschner P., Meier M.K., Wilken F., Rieger J., Beckmann J. (2023). Patient specific instruments and patient individual Implants—A narrative review. J Personalized Med.

[3] Bodendorfer B.M., Loughran G.J., Looney A.M., Velott A.T., Stein J.A., Lutton D.M. (2021). Short-term outcomes of reverse shoulder arthroplasty using a custom baseplate for severe glenoid deficiency. J Shoulder Elbow Surg.

[4] Burton R., Adam J., Holland P., Rangan A. (2023). A review of custom implants for glenoid bone deficiency in reverse shoulder arthroplasty. J Orthop.

[5] Chammaa R., Uri O., Lambert S. (2017). Primary shoulder arthroplasty using a custom-made hip-inspired implant for the treatment of advanced glenohumeral arthritis in the presence of severe glenoid bone loss. J Shoulder Elbow Surg.

[6] Debeer P., Berghs B., Pouliart N., Van den Bogaert G., Verhaegen F., Nijs S. (2019). Treatment of severe glenoid deficiencies in reverse shoulder arthroplasty: the Glenius Glenoid Reconstruction System experience. J Shoulder Elbow Surg.

[7] Gomes N.S. (2016). Patient-specific instrumentation for total shoulder arthroplasty. EFORT Open Rev.

[8] Grammont P., Trouilloud P., Laffay J., Deries X. (1987). Concept study and realization of a new total shoulder prosthesis. Rhumatologie.

[9] Gruber M.S., Schwarz T., Lindorfer M., Rittenschober F., Bischofreiter M., Hochreiter J. (2022). Clinical and radiological outcomes after total shoulder arthroplasty using custom-made glenoid components: a systematic review. J Clin Med.

[10] Hu H., Liu W., Zeng Q., Wang S., Zhang Z., Liu J. (2019). The personalized shoulder reconstruction assisted by 3D printing technology after resection of the proximal humerus tumours. Cancer Manag Res.

[11] Katz D., O'Toole G., Cogswell L., Sauzieres P., Valenti P. (2007). A history of the reverse shoulder prosthesis. Int J Shoulder Surg.

[12] Kozak T., Bauer S., Walch G., Al-karawi S., Blakeney W. (2021). An update on reverse total shoulder arthroplasty: current indications, new designs, same old problems. EFORT Open Rev.

[13] Ortmaier R., Wierer G., Gruber M.S. (2022). Functional and radiological outcomes after treatment with custom-made glenoid components in revision reverse shoulder arthroplasty. J Clin Med.

[14] Porcellini G., Micheloni G.M., Tarallo L., Paladini P., Merolla G., Catani F. (2021). Custom-made reverse shoulder arthroplasty for severe glenoid bone loss: review of the literature and our preliminary results. J Orthop Traumatol.

[15] Rangarajan R., Blout C.K., Patel V.V., Bastian S.A., Lee B.K., Itamura J.M. (2020). Early results of reverse total shoulder arthroplasty using a patient-matched glenoid implant for severe glenoid bone deficiency. J Shoulder Elbow Surg.

[16] Rashid M.S., Cunningham L., Shields D.W., Walton M.J., Monga P., Bale R.S. (2023). Clinical and radiologic outcomes of Lima ProMade custom 3D-printed glenoid components in primary and revision reverse total shoulder arthroplasty with severe glenoid bone loss: a minimum 2-year follow-up. J Shoulder Elbow Surg.

[17] Rolf O., Mauch F. (2020). Individualisierte Schulterendoprothetik: aktueller Stand der Entwicklung. Orthopade.

[18] Schroeder L., Dunaway A., Dunaway D. (2022). A comparison of clinical outcomes and implant preference of patients with bilateral TKA: one knee with a patient-specific and one knee with an off-the-shelf implant. JBJS Rev.

[19] Senthil V. (2023). 3D printing in arthroplasty: a systematic review. J Orthop Jt Surg.

[20] Sirveaux F., Favard L., Oudet D., Huquet D., Walch G., Molé D. (2004). Grammont inverted total shoulder arthroplasty in the treatment of glenohumeral osteoarthritis with massive rupture of the cuff. Results of a multicentre study of 80 shoulders. J Bone Joint Surg Br.

[21] Smith P.N., Gill D.R., McAuliffe M.J., McDougall C., Stoney J.D., Vertullo C.J. (2023). Hip, Knee and Shoulder Arthroplasty: 2023 Annual Report, Australian Orthopaedic Association National Joint Replacement Registry, AOA: Adelaide, South Australia.

[22] Walch G., Badet R., Boulahia A., Khoury A. (1999). Morphologic study of the glenoid in primary glenohumeral osteoarthritis. J Arthroplast.

