# Supplementary material for: Customized glenoid implants in reverse total shoulder arthroplasty: assessing revision rate and causes of failure in the Australian Joint Registry
Source: J Shoulder Elb Arthroplast. 2026 Sep 29;10(4):100098. doi: 10.1016/j.jsea.2026.100098 (PMC13734920; doi:10.1016/j.jsea.2026.100098)
Supplement: Supplementary file 1 [file mmc1.docx]

**Appendices**

**Table A.1.** Catalogue Numbers of Custom Glenoids included in analysis of revision rate of primary rTSA in AOANJRR cohort.

| **Model** | **Catalogue Range** | **Range Description** | **No. of Primary Procedures** |
| --- | --- | --- | --- |
| Custom Made (Comprehensive) | PM0000049 - PM0004549 | Custom Comprehensive Reverse Glenoid Baseplate | 60 |
| Custom Made (Comprehensive) | PM0000279 - PM0004145 | Custom Comprehensive Convertible Glenoid | 2 |
| Custom Made (Comprehensive) | PM555238 - PM555419 | Custom Comprehensive Reverse Glenoid Ti Porous Bonemaster HA | 11 |
| Custom Made (Comprehensive) | PM555355 - PM555422 | Custom Comprehensive Convertible Glenoid | 1 |
| Custom Made (Lima) | 961714051 - 961714389 | Custom Made Augmented Glenoid Ti6Al4V | 6 |
| Custom Made (Lima) | 961714410 - 96181600C | Custom Made Glenoid Implant Ti6Al4V | 37 |
| Custom Made (Lima) | 961714A02 - 9618140NH | Custom Made Glenoid Implant Ti6Al4V | 21 |
| Custom Made (Materialise) | MOBGR10 - MOBGR000 | Custom Made Glenoid Ti6Al4V | 7 |
| Custom Made (Mobelife) | HEMSH201511101A199 - HEMSH201511101A199 | Custom made Metal Backing Glenoid | 1 |
| Custom Made (Mobelife) | HP201609271A199 - HP201609271A299 | Custom Made Glenoid | 1 |
| Custom Made (Mobelife) | STRC201511181A199 - STRC201511181A199 | Custom made Metal Backing Glenoid Ti | 1 |
| Custom Made (Signature) | 141021338 - 141021941 | Custom Glenoid Reverse Baseplate | 5 |
| Trabecular Metal | 32855090250 - 32855090739 | Custom Made Reverse Base Plate | 4 |

**Table A.2.** Catalogue Numbers of Custom Glenoids included in analysis of revision rate of 1^st^ revision rTSA in AOANJRR cohort.

| **Model** | **Catalogue Range** | **Range Description** | **No. of Revision Procedures** |
| --- | --- | --- | --- |
| Aequalis | DXX002 - DXX002 | Custom Base Plate Ti HA | 1 |
| Custom Made (Comprehensive) | PM0000049 - PM0004549 | Custom Comprehensive Reverse Glenoid Baseplate | 13 |
| Custom Made (Comprehensive) | PM0000279 - PM0004145 | Custom Comprehensive Convertible Glenoid | 1 |
| Custom Made (Comprehensive) | PM555355 - PM555422 | Custom Comprehensive Convertible Glenoid | 1 |
| Custom Made (Lima) | 961714051 - 961714389 | Custom Made Augmented Glenoid Ti6Al4V | 3 |
| Custom Made (Lima) | 961714410 - 96181600C | Custom Made Glenoid Implant Ti6Al4V | 4 |
| Custom Made (Lima) | 961714A02 - 9618140NH | Custom Made Glenoid Implant Ti6Al4V | 4 |
| Custom Made (Lima) | 961725001 - 961725002 | Custom made Glenoid Long Peg MB S-R Ti6Al4V PoroTi HA | 2 |
| Trabecular Metal | 32855090250 - 32855090739 | Custom Made Reverse Base Plate | 2 |
